# In Search of New Therapeutics—Molecular Aspects of the PCOS Pathophysiology: Genetics, Hormones, Metabolism and Beyond

**DOI:** 10.3390/ijms21197054

**Published:** 2020-09-25

**Authors:** Agata Wawrzkiewicz-Jałowiecka, Karolina Kowalczyk, Paulina Trybek, Tomasz Jarosz, Patrycja Radosz, Marcin Setlak, Paweł Madej

**Affiliations:** 1 Department of Physical Chemistry and Technology of Polymers, Silesian University of Technology, 44-100 Gliwice, Poland; Tomasz.Jarosz@polsl.pl; 2 Department of Obstetrics and Gynecology, Medical University of Silesia in Katowice, 40-752 Katowice, Poland; karolina.kowalczyk@sum.edu.pl (K.K.); patrycja_radosz1@wp.pl (P.R.); pmadej@sum.edu.pl (P.M.); 3 Faculty of Science and Technology, University of Silesia in Katowice, 41-500 Chorzow, Poland; paulina.trybek@smcebi.edu.pl; 4 Department of Neurosurgery, Medical University of Silesia, 40-752 Katowice, Poland; 7marcin.setlak7@gmail.com

**Keywords:** polycystic ovary syndrome (PCOS), molecular mechanism, novel therapies, inositols, GABA, kisspeptin, berberine, naringenin, AQPs-oriented therapy

## Abstract

In a healthy female reproductive system, a subtle hormonal and metabolic dance leads to repetitive cyclic changes in the ovaries and uterus, which make an effective ovulation and potential implantation of an embryo possible. However, that is not so in the case of polycystic ovary syndrome (PCOS), in which case the central mechanism responsible for entraining hormonal and metabolic rhythms during the menstrual cycle is notably disrupted. In this review we provide a detailed description of the possible scenario of PCOS pathogenesis. We begin from the analysis of how a set of genetic disorders related to PCOS leads to particular malfunctions at a molecular level (e.g., increased enzyme activities of cytochrome P450 (CYP) type 17A1 (17α-hydroxylase), 3β-HSD type II and CYP type 11A1 (side-chain cleavage enzyme) in theca cells, or changes in the expression of aquaporins in granulosa cells) and discuss further cellular- and tissue-level consequences (e.g., anovulation, elevated levels of the advanced glycation end products in ovaries), which in turn lead to the observed subsequent systemic symptoms. Since gene-editing therapy is currently out of reach, herein special emphasis is placed on discussing what kinds of drug targets and which potentially active substances seem promising for an effective medication, acting on the primary causes of PCOS on a molecular level.

## 1. Introduction

Polycystic ovary syndrome (PCOS) is the most common endocrinopathy of premenopausal women [1]. The medical and scientific understanding of the etiology of PCOS remains incomplete, even though it already embraces a complex mixture of genetic and epigenetic factors [2,3,4,5,6]. This heterogeneous disease is diagnosed based on the occurrence of the following symptoms (either all, or at least some of them): menstrual cycle irregularities (amenorrhea or oligomenorrhea); androgen excess; anovulations resulting in infertility and polycystic ovaries, as visualized by ultrasonography [1,7]. Additionally, hyperandrogenism can lead to, e.g., hirsutism, acne and androgenic alopecia. The majority of PCOS cases are accompanied by metabolic abnormalities, such as obesity, insulin resistance, hyperinsulinemia, dyslipidemia or elevated levels of the advanced glycation end products [8,9].

The most common treatment schemes for polycystic ovary syndrome involve recommendations of lifestyle changes (particularly diet and enhanced physical activity), oral contraceptives and metformin, applied in order to restore regular menstruation, lower insulin resistance level and retain a normal body weight [10,11,12]. Additionally, letrozole and clomiphene citrate are frequently used to induce ovulation [13,14,15].

That kind of therapy is mainly focused on treating the symptoms and in some cases fails to yield satisfactory results. In the case of this complex disease, which affects a broad range of systems within the organism, it appears that the mutual interactions of the malfunctioning systems can prevent therapy from being effective. From the perspective of clinical practice, the most problematic issues, which occur despite the application of conventional medications, are:

1. Difficulties in inducing ovulation in some patients, despite stimulation by pharmacological agents (letrozole, clomiphene citrate, or gonadotropins).
2. Unsatisfactory reduction of insulin resistance and hyperinsulinemia, in spite of implementing lifestyle changes and undergoing metformin therapy.
3. Frequent unsatisfactory results of attempts to counteract hirsutism, acne and androgenic alopecia by application of, e.g., oral Contraceptives—thus, the sensitivity of androgen receptors within the skin to testosterone and dihydrotestosterone (DHT) should be lowered.

**Figure 1:**
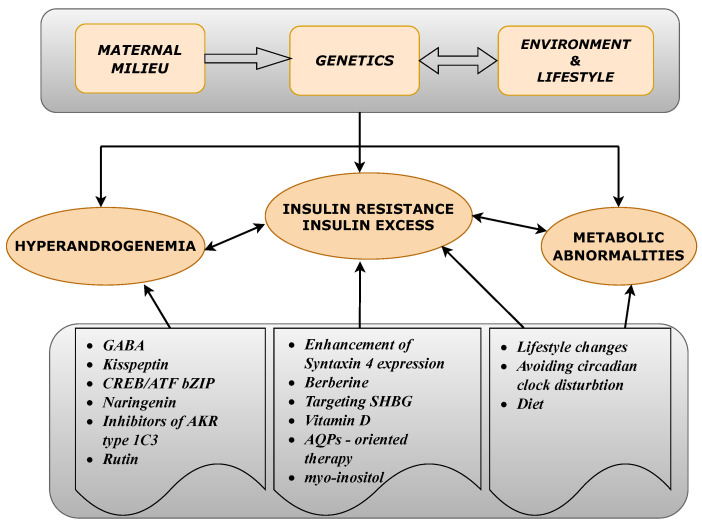
A conceptual diagram illustrating the basic factors contributing to the main polycystic ovary syndrome (PCOS)-related disorders and the proposed therapeutics either based on the potentially effective active substances or acting on recently proposed drug targets.

These and many other PCOS-related problems have to be addressed, in order to improve the medical procedures employed against PCOS. In this work we investigate the pathogenesis of PCOS in a step-by-step manner, paying special attention to both the genetic background and also epigenetic factors leading to the molecular defects related to PCOS. The cause and effect relationships within the biological mechanisms leading to this endocrinopathy are emphasized. From such a perspective, we summarize the most promising scientific developments in the search for novel drug targets, and active substances, which can act on the initial causes of PCOS at the molecular level. The main idea of this work was summarized in a form of a conceptual diagram (Figure 1).

## 2. Leading Factors in PCOS Pathogenesis

In this Section we examine the main factors leading to the development of polycystic ovary syndrome, starting from genetic and developmental factors, and investigate the resulting disturbances from the molecular level to the peripheral consequences.

### 2.1. Developmental Programming of PCOS

It appears that PCOS is one of the diseases in which exposure to environmental factors, occurring during gestation and the early postnatal period, causes alterations of gene expression patterns, consequently enhancing susceptibility to that disorder later in life. According to clinical and pre-clinical studies, women with PCOS tend to have elevated intrauterine androgen levels. Congenital adrenal hyperplasia is one such medical condition that causes high prenatal adrenal exposure. Due to hyperandrogenemia during pregnancy, the offspring is more likely to develop PCOS [16].

In the past few years, many animal models involved implementing exogenous androgens, in order to examine the role of developmental factors in PCOS pathogenesis [17]. One of the new animal PCOS models involved mice that were exposed prenatally to an excess of anti-müllerian hormone (AMH). This hormone is constantly elevated in PCOS-afflicted women, including during pregnancy. Long-lasting increase of AMH levels in women afflicted by PCOS is attributed to abnormally high antral follicle recruitment during folliculogenesis. Female mice exposed to AMH developed increased activity of gonadotropin-releasing hormone (GnRH) neurons and, as a result, frequent luteinizing hormone (LH) pulses and increased androgen levels. AMH acted centrally by inducing GnRH neuronal activation, which finally resulted in hyperandrogenemia affecting both mother and fetus. Female offspring that developed in the environment of increased androgen level, presented a PCOS phenotype in adulthood. A very promising fact concerning novel therapies is that GnRH antagonist treatment in the adult female offspring restored their neuroendocrine phenotype to a normal state. However, this kind of study should be conducted more, in order to determine whether models with both exogenous androgens and AMH administration act on the same cellular and molecular pathways [16,18].

In another study, rodents were postnatally treated with exogenous androgens, especially dihydrotestosterone (DHT). In adolescence, they presented a PCOS phenotype, with metabolic symptoms and polycystic ovaries, but they did not present increased LH secretion. Conversely, they had reduced LH levels in comparison with other animal models and PCOS-afflicted women. The hyper-activation of hypothalamic-pituitary functioning in these groups is a focal point that remains to be studied in more detail in the future [16,19].

### 2.2. Genetics in PCOS

It is extremely difficult to provide a comprehensive and precise report about the genetic basis of a highly polygenic and multifactorial disorder like PCOS. Due to its complex etiology, including endocrine disturbances, insulin resistance, inflammation, etc., the majority of genes that have any impact on the functioning of ovaries can be involved in the development of PCOS. In addition, the existence of different PCOS phenotypes makes genomic research more complex and difficult to elucidate. Nevertheless, in this section we summarize the proven results showing direct links between some particular alterations in the genome and their PCOS-related consequences. The roles of different gene types are frequently divided into several groups: ovarian and adrenal steroidogenesis, genes involved in steroid hormone actions, gonadotropin action and regulation, genes involved in insulin action and secretion, genes with impacts on energy homeostasis and genes involved in chronic inflammation [4,5,20]. Table 1 summarizes the most important genes associated with the occurrence of PCOS.

### 2.3. Hyperandrogenemia

Hyperandrogenemia is an essential symptom of PCOS. Androgens are produced in the ovaries and adrenal glands as the final products of a series of enzymatic reactions starting from a common precursor, i.e., cholesterol. The critical intermediate stages of androgen production involve the conversion of cholesterol into dehydroepiandrosterone and androstenedione. These reactions take place in the theca cells (in the ovary) and in the adrenal cortex (in the adrenal gland). In both locations, the rate of sex steroid synthesis is limited by some crucial enzymes. As described in the previous section, the over-expressions of particular genes lead the molecular characterization of PCOS, which involves increased expression of the following steroidogenic enzymes of the cytochrome P450 (CYP), hydroxysteroid dehydrogenase (HSD) and aldo-keto reductase (AKR) families: CYP type 17A1 (17α-hydroxylase), CYP type 11A (cholesterol side-chain cleavage), 3β-HSD type II and AKR type 1C1 (20a-hydroxysteroid reductase) [41,42]. Moreover, the activity of enzymes (mainly CYP type 17A1) involved in androgen biosynthesis is dose-dependently regulated by LH in the ovary and by adrenocorticotropic hormone in the adrenal cortex [8]. High availability of steroidogenic enzymes and an elevated level of LH determines fast androgen biosynthesis.

**Figure 2:**
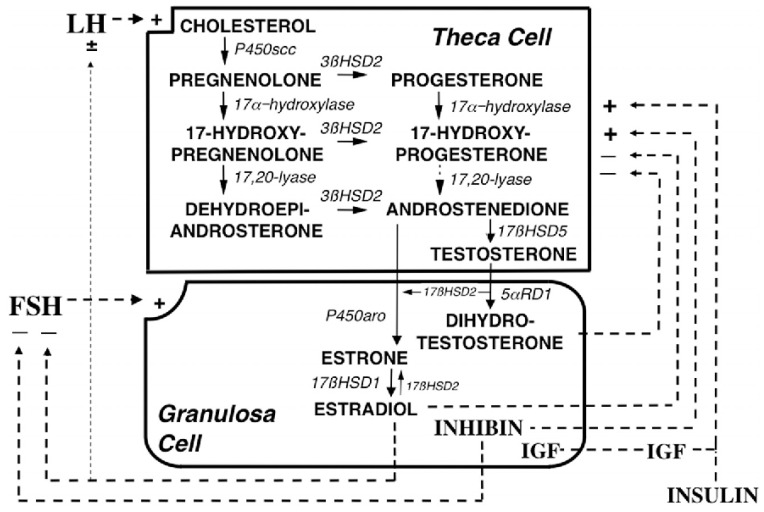
Depiction of the organization and regulation of the major steroid biosynthetic pathways in the small antral follicle of the ovary according to the 2-gonadotropin, 2-cell model of ovarian steroidogenesis, taken and captioned from [42] with permission of the Oxford University Press. LH stimulates androgen formation within theca cells via the steroidogenic pathway common to the gonads and adrenal glands. FSH regulates estradiol biosynthesis from androgen by granulosa cells. Long-loop negative feedback of estradiol on gonadotropin secretion does not readily suppress LH at physiological levels of estradiol and stimulates LH under certain circumstances. Androgen formation in response to LH appears to be modulated by intraovarian feedback to the levels of 17-hydroxylase and 17,20-lyase, both of which are activities of CYP type 17A1, which is expressed only in theca cells. The relative quantity of androstenedione formation via 17OHP (dotted arrow) in the intact follicle is probably small, as is the amount of progesterone formed from granulosa cell CYP type 11A activity in response to FSH (data not shown). Additionally, 17 HSD2 activity is minor in the ovary, and estradiol is primarily formed from androstenedione. Androgens and estradiol inhibit (minus signs), and inhibin, insulin and IGF-1 (IGF) stimulate (plus signs) 17-hydroxylase and 17,20-lyase activities.

Testosterone is further converted into estrogen in granulosa cells and this reaction is mediated by CYP type 19A1 (also referred to as estrogen synthetase, as it is also involved in the direct conversion of androstenedione into estrone, that is further metabolized to estradiol by 17β-HSD type I). Transcription of the CYP type 19A1 (estrogen synthetase) gene is regulated by follicle stimulating hormone (FSH). Thus, an elevated LH/FSH ratio in PCOS is partially responsible for the hyperandrogenism in this syndrome. The detailed description of steroidogenesis is given in [42], and summarized in Figure 2.

In recent years, researchers have demonstrated a strong association between LH gene polymorphism and PCOS in women. Further studies also identified genes in or near the LH/choriogonadotropin receptor and FSH receptor and variant in the FSHβ gene (Table 1). The mutation of FSHβ resulted in the decrease of the FSH level, increase in the LH level and impaired folliculogenesis. It has been shown that these genes may be responsible for the pathogenesis of PCOS [43].

As mentioned before, hyperandrogenemia in PCOS is caused in part by the elevated level of LH and one can also observe its increased pulsatile secretion from the pituitary in PCOS patients. The occurrence of frequent high pulses of LH depends on the preceding secretion of GnRH. That is why many studies emphasize the neuroendocrine basis of PCOS [42].

According to animal studies, AMH, which is commonly elevated in women with PCOS (due to excessive accumulation of small antral follicles in their ovaries), may stimulate GnRH neuron activity since these neurons express AMH type II receptors [18]. Thus, high levels of AMH may enhance secretion of gonadotropin-releasing hormone and support excessive androgen production.

Many neural and endocrine factors also influence the secretion of GnRH from the brain. γ-Aminobutyric acid (GABA) and kisspeptin expressed by the hypothalamic neurons are some of these factors. The afferent regulation of GnRH neurons in PCOS is still not fully understood, though there is high probability it constitutes the basis of GnRH and LH hypersecretion in this syndrome [44].

An excess of androgens in PCOS leads to the development of metabolic complications related to this syndrome [8], including global adiposity, adipocyte hypertrophy and adipocyte dysfunction (e.g., impaired insulin sensitivity), eventually leading to the observed central obesity and insulin resistance. In particular, in fat adipocytes during lipolysis, lipid triglycerides are hydrolyzed into glycerol and free fatty acids (FFA). Androgens can downregulate hormone-sensitive lipase (HSL) and β-2 adrenergic receptor expression, and consequently reduce catecholamine-stimulated lipolysis, particularly in subcutaneous adipocytes [45]. An additional effect may be exerted by the underexpression of aquaporin 7 (AQP7) in adipocytes, as a consequence of human AQP7 genetic polymorphism (the A-953G SNP causing AQP7 down-regulation), which is related to obesity [46]. AQP7 is a glycerol channel, responsible for the efflux of glycerol and FFAs from fat cells into the bloodstream. AQP7 deficiency results in reduced membrane glycerol permeability which leads to increased glycerol concentration inside adipocytes and increased activity of glycerol-3-phosphate. In those conditions, re-esterification of FFAs becomes preferential. The aforementioned processes end with progressive triacylglycerol accumulation and adipocyte hypertrophy. The excess adiposity sustains a state of chronic low-grade inflammation (release of inflammatory cytokines and chemokines), which leads to further insulin resistance [47,48].

The insulin sensitivity of skeletal muscles is also affected in hyperandrogenic women with PCOS. Namely, the literature [8,49] indicates alterations in the insulin-signaling pathway (decreased tyrosine phosphorylation and increased serine phosphorylation of the insulin receptor), and disturbances of the intracellular insulin pathway (reduced Akt/PKB and AS160 phosphorylation and activation in muscle cells, which impair insulin-stimulated glucose uptake and therefore contribute to insulin resistance).

An excess of testosterone in PCOS patients exerts an additional effect, because it interferes with insulin signalling in peripheral tissues. In adipose tissue, testosterone acts via decreased protein kinase C, and in skeletal muscles, it acts via increased mammalian target of rapamycin and ribosomal S6-kinase [50].

Brown adipose tissue (BAT), which is responsible for adaptive thermogenesis, produces heat due to the presence of increased levels of uncoupling protein 1 (UCP-1) within the mitochondria, which promotes heat production by uncoupling aerobic respiration. It has been shown that women with PCOS exhibit decreased BAT functionality resulting from high androgen levels (testosterone reduces UCP-1 levels) [51]. The animal models using rats suggest that there may be a link among BAT activity, circulating adiponectin levels and the development of PCOS [52].

One of the most prominent consequences of hyperandrogenemia is arrested antral follicle development and anovulation. In the search for the exact mechanism of anovulation induced by androgen excess, a mouse model of PCOS was investigated. The authors concluded that over-activated C-type natriuretic peptide (CNP) and natriuretic peptide receptor 2 (CNP/NPR2) system plays an important role in oocyte meiotic arrest and preventing ovulation. It turned out that an effective treatment included administration of human chorionic gonadotropin (hCG) or inhibitors of AR or ER, which reduced the level of CNP/NPR2 and restored oocyte maturation and ovulation [53]. Since CNP is involved in regulating gonadal and reproductive functions, a further investigation of the underlying mechanism in humans is needed from the perspective of future fertility therapies, and in PCOS [53,54].

An additional problem, contributing to abnormal steroidogenesis and folliculogenesis in women with PCOS, is the elevated level of harmful advanced glycation end products (AGEs), which are accumulated in the ovaries [9,55]. AGEs, also referred to as “glycotoxins”, are highly reactive molecules formed after the glycation of lipids and proteins as the byproducts of the Maillard reaction. Recent reports state that AGEs are involved in excessive androgen production in PCOS, because they modulate the activities of crucial steroidogenesis enzymes, such as: CYP type 11A, CYP type 17A1 and 3β-HSD [56,57]. AGEs may interfere with intracellular insulin signaling and glucose transport in human granulosa cells, potentially affecting ovarian function and follicular growth [58]. AGEs also affect LH receptor and AMH receptor expression, and their signaling pathways in granulosa cells [9].

### 2.4. Insulin Resistance and Hyperinsulinemia

**Figure 3:**
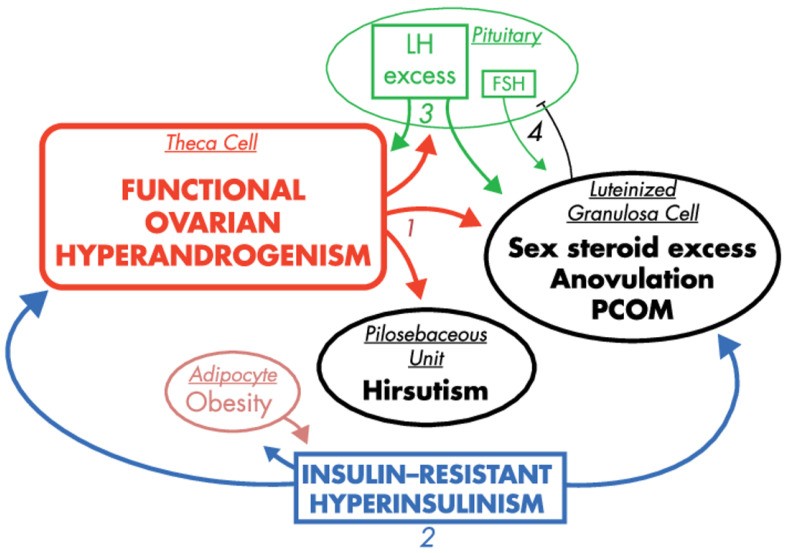
The effects of hyperinsulinemia in the context of PCOS pathogenesis, reprinted and captioned from [42] with permission of the Oxford University Press. Ovarian hyperandrogenism is nearly universal in PCOS and can account for all the cardinal clinical features of the syndrome: hyperandrogenemia, oligo-anovulation and polycystic ovaries (1). About half of patients with functional ovarian hyperandrogenism have insulin-resistant hyperinsulinism (2). Insulin-resistant hyperinsulinism acts on theca cells to aggravate hyperandrogenism, synergizes with androgen to prematurely luteinize granulosa cells and stimulates adipogenesis. The increased hyperandrogenemia provokes LH excess (3), which then acts on both theca and luteinized granulosa cells to worsen hyperandrogenism. LH also stimulates luteinized granulosa cells to secrete estradiol (4), which suppresses FSH secretion. These hyperinsulinism-initiated changes in granulosa cell function further exacerbate PCOS and further hinder ovulation. Obesity increases insulin resistance, and the resultantly increased hyperinsulinism further aggravates hyperandrogenism. Heaviness of lines and fonts represents severity. Both functional ovarian hyperandrogenism and insulin resistance typically have an intrinsic basis. This model does not exclude the possibility that the unknown intrinsic ovarian defects that underpin the ovarian steroidogenic dysfunction also involve granulosa cell folliculogenesis. The figure also does not depict other associated defects, such as the functional adrenal hyperandrogenism that often accompanies the ovarian hyperandrogenism and the contribution of excess adiposity to peripheral androgen production and gonadotropin suppression.

The majority of women with PCOS exhibit insulin resistance. This symptom is further exacerbated in obese PCOS patients as a result of their excess adiposity. The forthcoming consequence of the insulin resistance is hyperinsulinemia, which stems in part from excess release of insulin in the pancreas to compensate for insulin resistance. In turn, biosynthesis of sex steroids is modulated by insulin and insulin-like growth factor-1 (IGF-1), which are considered as most notable extra-ovarian factors stimulating androgen production. Both substances support ovarian and adrenal steroidogenesis (by increasing the activity of CYP type 17A1, or activating steroidogenic factor (SF-1) and its steroidogenic target genes, respectively) [42,59]. Additionally, insulin also modulates LH pulse amplitude [60], and suppresses hepatic SHBG production [61]. From this perspective, insulin resistance and hyperinsulinemia seem to be the most prominent extra-ovarian factors imposing symptoms of PCOS [62], as presented in Figure 3.

An interesting question is whether insulin is directly involved in the arrest of preantral and antral follicle development, and another is what the underlying molecular mechanism is. The local expressions of IGFs, their receptors, IGF binding proteins (IGFBPs) and IGFBP proteases are undoubtedly important in normal and abnormal ovarian follicle development, as confirmed by the animal models [63,64,65]. However, unraveling the exact molecular mechanism indicating how insulin can interfere with the growing follicle, along with its role in ovulation, still remains a task for future research.

### 2.5. Environmental Factors

The issue of the impacts of various environmental factors on PCOS pathogenesis has recently been the subject of several reviews [66], with numerous factors, such as exposure to environmental toxins, diet and nutrition [67]; and socioeconomic status and geography, having been discussed in detail. Consequently, only the most relevant recent advances are mentioned herein.

One such environmental factor is the disruption of the circadian cycle [68], which has for some time been suspected to be a contributing factor for PCOS pathogenesis, but has only recently been investigated experimentally [69]—prolonged exposure to continuous light was found to induce several PCOS-like changes in rats; i.e., the ovarian morphology showed abundant atretic cyst-like follicles and reduced the number of corpora lutea, demonstrating oligo/anovulation. AMH levels were significantly elevated in comparison with the control groups; the glucose metabolism, evidenced by a lower basal insulin level and decreased homeostatic model assessment, was also impaired.

Exposure to fluorine may be another potential environmental risk factor, even at concentrations considered to be normal. A recent work [70] reports that increased TSH levels (even though still within the range considered to be normal) were found for women with higher fluorine concentrations in plasma. Simultaneously, however, the insulin resistance index was significantly higher and indicative of advanced insulin resistance in this group, as opposed to both the control group and for women with low plasma fluorine concentrations. Consequently, this increased insulin resistance translated to reduced SHBG synthesis.

Among environmental factors, exposure to substances classified as endocrine disruptors is also relevant to the pathogenesis of PCOS. Such substances are commonly encountered in our surroundings, being found in particular abundance and variety in consumer plastics, with the adverse effects not being limited to PCOS pathogenesis [71]. Among such endocrine disruptors, bisphenol A [72], along with organochlorine pesticides, both of which can mimic the action of estrogen, can be considered the “flagship” compounds, to which particular scientific attention has been devoted.

Perfluoro-compounds, such as perfluorooctanoate, are also suspected to contribute to PCOS pathogenesis, as they have been found, at elevated levels, in the sera of PCOS-afflicted women [73].

Endocrine disruptors can also play a role through prenatal exposure. Studies on rodents revealed that gestational exposure to phthalates (dibutyl phthalate and di(2-ethylhexyl)phthalate) results in PCOS-like characteristics—polycystic ovaries and a hormonal profile similar to PCOS [74]. The same study has also associated exposure to nicotine and 3,4,4’-trichlorocarbanilide with the evolution of a hyperandrogenic fetal environment.

## 3. Potential Drug Targets and Active Substances in Novel Therapies

### 3.1. Attenuation of Developmental Programming of PCOS

The developmental programming of PCOS that causes the characteristic endocrine and metabolic changes in neonates might be modified postnatally by a so-called “second hit” in newborns [75]. The thorough analysis of the hormonal imbalance occurring prenatally may allow one to indicate what kinds of modifications to the medical care of women afflicted by PCOS should be introduced during their pregnancy to avoid development of the PCOS in their offspring.

In animal models, testosterone was implemented as a perinatal treatment, to analyze the development of PCOS. Especially in sheep and macaques, this pharmacotherapy during pregnancy resulted in the adult onset of PCOS in their offspring. Furthermore, the neuroendocrine, ovarian and metabolic imbalances caused by this therapy are similar to those observed in women afflicted by PCOS. There are not only changes in the reproductive system but also in the metabolic system. All of these results of animal models indicate that there should be strategies aimed simultaneously at multiple organs, in order to attenuate the developmental programming of PCOS [76].

Until now, no adequate treatment has been developed to prevent these developmental changes. Further studies should be conducted to find out whether any supplementation during pregnancy or any postnatal pharmacotherapy could attenuate the development of PCOS in the offspring. Animal models indicate that pharmacological and transgenic approaches to this topic can help to elucidate what molecular and genetic pathways are involved in developmental programming and to establish appropriate modern therapies. The aforementioned GnRH antagonists which restored normal GnRH frequency in mice are among possible candidates.

### 3.2. Approaches to Alleviate Hyperandrogenism

The pivotal point of effective PCOS treatment is to lower the circulating testosterone and androstenedione levels. To accomplish that goal, several strategies can be proposed, including: modification of the susceptibility of theca cells to LH stimulation; attenuation of key enzymes involved in androgen production levels (e.g., CYP type 17A1, CYP type 11A1, 3β-HSD type II, 17β-HSD and AKR type 1C3); reduction of pulse frequency of GnRH that could hamper LH production and balance the LH/FSH ratio; downregulation of GnRH gene expression in hypothalamic GnRH neurons; and attenuation of anti-müllerian hormone level. Below, we summarize the most promising scientific achievements which can offer good bases for novel therapies.

γ-Aminobutyric acid (GABA) has a stimulating effect on GnRH neurons. Increased levels of GABA have been detected in the cerebrospinal fluid of women with PCOS. Possibly, a high concentration of GABA in the cerebrospinal fluid of these women may increase the secretion of GnRH [77]. This observation was confirmed by studies conducted on mice. The results indicate the possibility that GABA neurons have an influence on the hyperactivity of GnRH neurons in PCOS-afflicted women, and in animal models. Furthermore, in animal models, the self-regulation of GABA neurons is also disturbed. The decrease in the expression of progesterone receptor was observed [78]. This finding of the impaired progesterone feedback was shown in the study, in which the women afflicted by PCOS required higher levels of progesterone to decrease the LH level, as compared to the women without PCOS [79].

Kisspeptin is a protein that potentially stimulates GnRH neurons. Recent studies showed a positive correlation between kisspeptin levels in PCOS patients and high LH levels in these women. The source of kisspeptin in PCOS remains unclear. This kind of correlation and the possibility that kisspeptin stimulates GnRH neurons makes kisspeptin an interesting point for further research. Kisspeptin and its membrane receptor Kiss1r signaling might potentially influence neural mechanisms in PCOS and be a new direction in the therapy of this disorder [16,80].

Some of the significant enzymes related to androgen production are CYP type 17A1, CYP type 11A1, 3β-HSD-2, 17β-HSD type V and AKR type 1C3. These enzymes are involved in the biosynthesis of androgens in adrenals and gonads. Recent studies have focused on their role in polycystic ovary syndrome [81]. CREB/ATF bZIP transcription factor is a protein in human organism that is encoded by the CREBZF gene. When lentiviral-mediated snRNA-CREBZF was injected into the mature mouse testis, the serum testosterone concentration was significantly decreased. What is more, silencing of CREBZF also decreased CYP type 17A1, 3β-HSD and 17β-HSD expression. This effect was reversed by the over-expression of CREBZF. These results show that the testosterone production, induced in male mice by human chorionic gonadotropin (hCG), is influenced by CREBZF. The expressions of 3β-HSD, 17β-HSD and CYP type 17A1 increase, which means that CREBZF is an important factor in the testosterone synthesis. Moreover, a natural substance—a flavanone from grapefruit, naringenin—is responsible for the reduction of the steroid enzymes (3β-HSD and 17β-HSD) activity in the PCOS rat model [82].

Melatonin receptors 1A and 1B regulate melatonin-mediated circadian rhythms, and the immune and reproductive systems in some animals. These receptors are expressed in the Leydig cell membrane and participate in the hCG-induced testosterone synthesis. When the melatonin receptors, especially receptor 1A, were knocked down, the decrease of the testosterone levels was observed. There was also a decrease of the steroidogenic gene expression—CYP type 11A1 and type 17A1 [83].

Aldo-keto reductase (AKR) type 1C3 is an enzyme that converts androstenedione to testosterone. It is highly expressed in the subcutaneous fat tissue in PCOS women and in obese women. When the androgen concentrations in systemic and adipose tissue were analyzed, it was proven that the androgen synthesis in PCOS was significantly expressed. Moreover, the activity of the AKR type 1C3 enzyme was also increased in these patients. The in vitro inhibition of AKR type 1C3 decreased androgen excess and de novo lipogenesis. Consequently, AKR type 1C should be considered as a new potential target for the treatment of PCOS [84].

The criteria for the diagnosis of polycystic ovary syndrome include an androgen excess confirmed by laboratory tests or resulting from clinical symptoms occurring in patients. Hyperandrogenemia, in addition to the obvious negative impact on the menstrual cycle, leads to hirsutism, alopecia and acne, which are serious problems for patients diagnosed with polycystic ovary syndrome [85,86]. Hirsutism is a problem that affects approximately 70% of women diagnosed with polycystic ovary syndrome and its occurrence is strongly associated with elevated androgen levels. In the case of acne and alopecia, this relationship is less clear and they are slightly less common than hirsutism [87]. It is well known that women diagnosed with polycystic ovary syndrome face higher levels of anxiety and are more likely to develop depressive disorders. Some researchers point to a direct relationship between these mood disorders and the occurrence of symptoms, such as acne or hirsutism, but opinions are divided on this issue [88,89,90,91,92]. Therefore, treatment should include counteracting all symptoms of the disease that are burdensome for patients [93].

The first line treatments for hirsutism, acne and alopecia in women (also PCOS patients) include cosmetic and combined oral contraceptive pills (COCPs). Since in some cases this kind of therapy gives unsatisfactory results, some trials were conducted through the use of anti-androgen (AA) pharmacological agents which were mostly combined with combined oral contraceptive pills (COCPs). Among the relatively widely tested AA substances applied in this kind of therapy, one can list finasteride and flutamide [94,95,96,97]. The mechanism of action of finasteride is based on its ability to inhibit 5α-reductase and consequently decrease the production of dihydrotestosterone (DHT) from testosterone and reduce the severity of hirsutism. Reducing the level of DHT is also beneficial in counteracting hair loss, because DHT can link to receptors on hair follicles in the scalp, causing them to shrink and become less capable of supporting hair. In turn, flutamide competes with testosterone and DHT for binding to androgen receptors (ARs), and thus acts as a selective antagonist of AR.

Nevertheless, the use of anti-androgens for treating alopecia and hirsutism remains controversial. Administration of anti-androgen agents (e.g., finasteride) brings teratogenic risks—thus, in that case the combined therapy with combined oral contraceptive pills (COCPs) appears to be necessary [95]. What is interesting is that even an intermittent low-dose administration of finasteride can be effective in counteracting hirsutism [95]. Due to specific flutamide-induced hepatotoxicity in women [96], administration of very low dosage was tested. This approach resulted in therapeutic success and proper hepatic enzyme levels being retained [97]. The reports presented hitherto show quite satisfactory results of such combined AA and COCP treatment; however, further research is needed to directly assess the therapeutic mechanisms and potential consequences of the use of anti-androgen pharmacological agents (finasteride, flutamide) and to optimize treatment protocols.

Another substance acting against androgenic alopecia is minoxidil [98], which can give particularly promising results without side-effects when applied at a relatively low concentration [99]. Minoxidil was first introduced as an antihypertensive medication, but it is also used as an off-label treatment for androgenic alopecia. Minoxidil is a potassium channel opener, and its positive effect on hair growth is mainly due to its metabolite, minoxidil sulfate. The enzyme responsible for this conversion is sulfotransferase, which is located in hair follicles. The molecular mechanism of minoxidil sulfate action on hair follicles seems to be relatively complex. Nevertheless, one of the leading component processes evoked by minoxidil is prolongation of the anagen phase in the dermal papilla (DP), by inducing β-catenin activity and stimulating follicular proliferation and differentiation [99,100].

The hormonal imbalance related to polycystic ovary syndrome is a common cause of female infertility, which has many negative consequences to physical health, psychological health and social well-being [101,102]. In women with PCOS, many therapeutic methods are used to increase their chances of getting pregnant. Research shows that lifestyle modifications and insulin resistance-counteracting substances are good initial treatments [103]. Drugs such as clomiphene citrate and gonadotropins are used to induce ovulation, but many patients require assisted reproductive techniques to get pregnant. Numerous studies are needed to thoroughly understand the mechanisms related to the maturation of ovarian follicles, which may allow the use of new substances in treatment [101,104].

The molecular mechanisms involved in oocyte meiosis have not been fully understood yet. Recent research conducted on mice shows that luteinizing hormone (LH)-induced downregulation of histone deacetylase 3 (HDAC3) plays an important role in the maturation process of oocytes [104]. The oocyte maturation process requires the proper functioning of granulosa cells within the ovarian follicle. Failures in the process of oocyte maturation and ovulation itself, which occur physiologically in response to the release of luteinizing hormone, most likely result from the inability to suppress the expression of certain genes in granulosa cells The study cited here demonstrated the importance of the mechanism of epigenetic modification in granulosa cells through histone acetylation by HDAC3 for gonadotropin-induced oocyte maturation. EGF-like growth factor mediates the effect of luteinizing hormone on oocyte maturation. The AREG protein, belonging to the EGF family, seems to be of greatest importance in this process. The use of AREG to improve the maturation of oocytes in vitro seems to be a candidate for an effective therapeutic option; however, numerous studies are necessary in this respect. Additionally, it was noticed that the luteinizing hormone release decreased the level of HDAC3, which was associated with an increase in AREG expression in granulosa cells. The use of an HDACi 4b inhibitor has also been associated with increased expression of AREG in vitro and in vivo, but the therapeutic use of an HDACi 4b inhibitor requires a great deal of research. The studies cited here have shown that pre-release, transcription factors cannot initiate AREG expression because the availability of the AREG promoter is blocked by HDAC3, and highlighted the key role of histone acetylation in specifically open and closed FSH and LH target genes in granular cells during oocyte maturation [104]. Another study conducted on animal models, and in women with PCOS, showed a significant influence of hyperandrogenism on epigenetic modifications leading to increased expressions of HDAC3 genes in granulosa cells in PCOS-afflicted women [105]. This study showed statistically significant differences in the level of HDAC3 expression between the study group (women with PCOS and hyperandrogenism) and the control group (women with PCOS without hyperandrogenism). Women with PCOS and hyperandrogenism showed higher HDAC3 expression than women with PCOS without hyperandrogenism. Moreover, a higher percentage of failed pregnancies was observed in women with PCOS accompanied by hyperandrogenism, as compared to the controls. Hyperandrogenism increases the expression of HDAC3 in granulosa cells, which causes ovarian dysfunction in women with PCOS [105]. These studies show that there are possibilities to influence the epigenetic processes related to oocyte maturation and ovulation in women with PCOS (e.g., by influencing AREG expression); however, careful research is needed in this area.

### 3.3. Sensitization to Insulin/Reduction of Hyperinsulinemia

As was mentioned above (Section *Insulin resistance and hyperinsulinemia*), insulin resistance is one of the basic symptoms of polycystic ovary syndrome. Hyperinsulinemia, which is associated with an increased risk of diabetes and cardiovascular diseases, appears to be secondary to tissue insulin resistance [11].

Apart from clomiphene and oral contraceptives, the mainstay of pharmacotherapy of polycystic ovary syndrome is metformin. Metformin is a substance with multiple sites of action and multiple molecular mechanisms of action. At the molecular level, the action of metformin is based on the inhibition of the respiratory chain in liver cells, which leads to the activation of adenosine monophosphate-activated protein kinase (AMPK), which, by influencing fat metabolism, increases insulin sensitivity and lowers cyclic adenosine monophosphate, which reduces the expression of gluconeogenic enzymes. Metformin also works by inhibiting fructose-1,6-bisphosphatase by adenosine monophosphate (AMP) [106]. Metformin has been used for many years as an agent to increase the tissue response to insulin in patients with type 2 diabetes. It has been used in patients with polycystic ovary syndrome for over 20 years. Research shows that metformin may find use as an ovulation stimulant in non-obese women with PCOS. Some researchers postulate that metformin may also be an effective agent in countering other symptoms that are associated with hyperandrogenism, including hirsutism and acne [107,108,109].

Proteins from the glucose transporter (GLUT) family are important for the efficient functioning of carbohydrate-related processes. These transmembrane glycoproteins are expressed in mammalian tissues and are responsible for the transport of glucose into the interior of cells, where it is used for the production of adenosine triphosphate and in numerous anabolic processes [110]. The disruption of a proper insulin stimulation that affects glucose transport to skeletal muscle cells and adipocytes via GLUT type 4 is the cause of peripheral insulin resistance that often occurs in PCOS [11,111,112]. Studies in animal models show that over-expression of syntaxin 4 (protein of the human body, encoded by the STX4 gene) may enhance the translocation of GLUT type 4 vesicles to the cell membranes of skeletal muscles and thus increase insulin sensitivity. In such a situation, the expression of Syntaxin 4 may provide a handle for new substances that could be used in people with insulin resistance, including women with PCOS [113,114,115,116].

Berberine is an interesting substance that seems to modulate GLUT type 4 expression. Berberine is an alkaloid that is found in many plants. Studies in rats show that it has beneficial results in normalizing peripheral insulin resistance by modulating GLUT type 4 expression [117].

Taking into account the notable role of insulin resistance in PCOS pathogenesis, the application of insulin sensitizer medicines, such as inositols, can be effective in the amelioration of PCOS symptoms [118,119,120,121]. Inositols are carbocyclic polyols. Myo-inositol (MI) and its stereoisomer D-chiro-inositol (DCI) are involved in insulin signaling when they are transformed to the form of inositol phosphoglycans (IPGs) [121]. MI is involved primarily in cellular glucose uptake, and thus, it is elevated in tissues with high glucose utilization and consumption, such as the brain and heart. Moreover, MI-IPG participates in the cellular uptake of glucose, inducing the GLUT4 translocation to the cell membrane. MI also inhibits adenylyl cyclase, and consequently reduces the release of free fatty acids from adipose tissues. In turn, DCI levels are high in tissues which store glycogen, such as liver, muscle and fat, and low in tissues with high glucose utilization. MI is converted in DCI by epimerase, an enzyme regulated also by insulin action [118]. In the ovary, MI regulates glucose uptake and follicle stimulating hormone (FSH) signaling, which affects ovulation. DCI directly regulates steroidogenesis by affecting the enzymes’ genes in human granulosa cells, thereby reducing the mRNA expression of both aromatase (CYP19A1) and cytochrome P450 side-chain cleavage (P450scc).

Considering the aforementioned physiological importance of inositols, a number of trials were performed to investigate the possibilities of administration of these compounds in PCOS treatment (their results were summarized in [121]). The best results were obtained when the MI and DCI were administered in a 40:1 ratio [119]. Such a ratio constitutes an appropriate approach to improving insulin sensitivity and ovulatory function, along with decreasing luteinizing hormone (LH) and free testosterone levels, which subsequently reduce the observed hyperandrogenism. The results of studies with myo-inositol were comparable to those obtained with metformin treatment, which makes it an effective and safe therapeutic option in women with PCOS suffering from insulin resistance [120]. Nevertheless, there still remain some unresolved problematic issues in inositol-based treatment of PCOS [121,122,123]. One of those issues is the therapeutic inefficacy of these compounds in some patients (in 30–40% of PCOS patients). It seems that this inositol “resistance” can stem from limited or lacking absorption of inositol. A possible way to overcome that problem is to combine the administration of MI with alpha-lactalbumin. Furthermore, however, the reports concerning fertility outcomes are conflicting [121,122]. From this perspective, further research is needed to optimize administration modes and the best combinations of MI, DCI and alpha-lactalbumin for different PCOS phenotypes.

As mentioned in previous sections, SHBG (sex hormone-binding globulin) seems to be an important therapeutic biomarker. Sex hormone-binding globulin is decreased in PCOS patients and appears to be associated with conditions such as hyperinsulinemia and insulin resistance [39]. Many strategies of treatment and potential drug-targets can be indicated regarding the aim of restoring a proper concentration of SHBG in PCOS patients. Below, we shortly discuss the most interesting aspects of SHBG regulation from the perspective of new medication developments against PCOS.

Chronic, low-grade inflammatory processes are often found in patients diagnosed with polycystic ovary syndrome [124]. Inflammatory processes are associated with excess androgens, insulin resistance, atherosclerosis and obesity. Patients with PCOS have elevated levels of C-reactive protein, interleukins and tumor necrosis factor-α, while SHBG levels are often lowered. This suggests that inflammatory cytokines may regulate SHBG expression, although the mechanism of this regulation has not been sufficiently understood.

Adiponectin and other adipokines [125] can be considered therapeutic targets for PCOS-related insulin resistance and obesity. Adiponectin plays an important role in counteracting the inflammatory process and increasing the sensitivity of tissues to insulin. Its decreased expression is believed to be one of the causes of hyperandrogenemia. Adiponectin lowers the level of liver lipids and increases the level of hepatocyte nuclear factor 4 alpha (HNF-4α) by activating AMPK. An increase in the level of HNF-4α promotes the synthesis of SHBG in the liver, while chronic inflammatory factors may indirectly reduce the level of HNF-4α, which results in a decrease in the production of SHBG and exposure to insulin resistance. It seems that systematically interfering with the emergence of the inflammatory processes occurring in the course of PCOS is an interesting therapeutic target.

Lifestyle change is a crucial part of treatment of women afflicted by PCOS. However, discussing this huge issue in detail remains beyond the scope of this review. Considering novel dietary approaches on the basis of recent studies in PCOS, it should be emphasized that a “modern” diet, including fast-foods and other highly processed foods that have been processed at high temperatures (baking, frying, or grilling), is also an important factor affecting PCOS traits. This kind of diet is an exogenous source of AGEs; thus, it directly correlates with high levels of androgens, AMH and insulin-promoting ovarian dysfunction and/or infertility [57,126,127].

### 3.4. Handling Other PCOS-Related Health Complications

It seems that ensuring the correct level of vitamin D may play a significant role in the treatment of symptoms associated with polycystic ovary syndrome. In studies conducted in rats and randomized studies conducted in women diagnosed with polycystic ovary syndrome, a greater thickness of the endometrium was found in females who had normal levels of vitamin D, which resulted in a greater chance of getting pregnant [128]. Additionally, vitamin D attenuates the effects of AGEs in women with PCOS (enhanced androgen synthesis, abnormal folliculogenesis) [57,126]. Namely, the addition of vitamin D3 to AGEs in vitro inhibited changes in the expression of these genes, possibly due to downregulation of RAGE mRNA and protein expression [57,126].

Some researchers point to aquaporins (AQPs) 7–9 as the targets of drugs that could be used in the treatment of PCOS [129]. The activity of these aquaporins depends on the levels of testosterone, estrogens and glycerol, which may be disturbed in the course of polycystic ovary syndrome. There is also evidence that aquaporins 7–9 are important in the construction of normal ovarian follicles that are disturbed in the course of PCOS.

The above is supported by a more recent study, which examined AQP 8 and 9 levels in the ovarian tissue and follicular fluid of PCOS patients. Elevated AQP 8 expression and decreased AQP 9 expression were observed and were correlated with the incidence of PCOS, leading to the idea of using the two AQPs as biological indicators of the condition [130]. The issue is touched upon in another study, focusing on AQP 7 and 9, although the expression of both AQPs was found to be elevated in the PCOS-afflicted group in comparison with the control group [131]. Incidentally, AQP 8 deficiency was found to lead to an increase in the formation of follicular antra via impaired proliferation of granulosa cells [132]. Although the trends found in the above studies differ, they can be interpreted as correlating AQP expression imbalances either directly with the occurrence of PCOS or with alterations to the formation of follicles. Correlation, by itself, cannot imply causation; nevertheless, perhaps investigating the origins of this AQP expression imbalance may shed new light on the causes of PCOS.

Another interesting substance that may be useful in fighting PCOS symptoms is naringenin. Naringenin is a substance of plant origin found in grapefruit and belongs to the group of natural flavanones. A study in rats has shown that it may be effective in controlling the symptoms of PCOS by preventing the weight gain associated with this disorder, and it caused a decrease in the serum glucose levels of PCOS rats [133]. Naringenin in a rat model showed a decrease in the activity of the enzymes 3β-hydroxysteroid dehydrogenase (3β-HSD) and 17β-hydroxysteroid dehydrogenase (17β-HSD) (as mentioned before in Section Approaches to alleviate hyperandrogenism), probably due to the presence of the B ring of the naringenin molecule. Earlier research has also shown that naringenin can reduce testosterone and estradiol levels in women with polycystic ovary syndrome. The conducted research has also shown that naringenin increasing the concentrations of enzymes removing reactive oxygen species and reducing oxidative stress is one of the characteristics of polycystic ovary syndrome. The cytoprotective and anti-inflammatory effects of naringenin were also described in [134], where the authors described the naringenin-mediated protection of cells against damage induced by TNF-α in combination with cycloheximide in an in vitro experiment. The described properties of naringenin justify considering it as an element of PCOS therapy [133].

What is worth mentioning is that naringenin is not the only flavonoid found in a wide variety of plants that can be helpful in PCOS therapy. Namely, promising results were obtained by intervention with rutin in rats [135]. Rutin promotes activation of brown adipose tissue, and in this way ameliorates metabolic and reproductive phenotypes in DHEA-induced PCOS rats. This kind of strategy shows the therapeutic potential of BAT-activating compounds for the treatment of PCOS.

A potential molecule for PCOS treatment may be also anethole, found in essential oils, as suggested by an in silica study, which predicted it to show a greater binding for androgen receptors than that of FSH receptors and IRS 1 receptors [136].

## 4. Discussion

Polycystic ovary syndrome is a complex diagnostic and therapeutic problem that affects many women of reproductive age. Due to the complex etiology of this disease, all the molecular mechanisms involved in the development of the polycystic ovarian syndrome phenotype have not been fully elucidated [85,86,87]. Moreover, even the course of the illness implies the occurrence and enhancement of some symptoms (like in the case of interrelation of insulin and androgen levels), which impedes efficient treatment.

The available literature clearly indicates what kind of problems women with PCOS face. Difficulties with getting pregnant, along with features related to hyperandrogenism, such as hirsutism or acne, may adversely affect the mental condition of these women and be associated with a higher feeling of anxiety or the risk of developing depression [88,89,90,91]. Interestingly, new studies conducted in animal models indicate a greater risk of developing the PCOS phenotype among female offspring of women with polycystic ovary syndrome [16,75]. In light of these facts, it seems obvious how much importance should be attached to a full understanding of the molecular mechanisms involved in the development of the PCOS phenotype. Detailed knowledge of the pathomechanism of this disease will probably allow one to select the target points for new substances that help counteract hyperandrogenization and the development of insulin resistance [85,86,87]. An interesting place for the action of new drugs seems to be the hypothalamic-pituitary axis. GnRH, which stimulates the anterior pituitary gland into pulsatile secretion of FSH and LH, which are responsible for the production of estrogens and androgens by the cells of the ovary, can be stimulated by substances such as GABA and kisspeptin. An excess of LH and an improper FSH/LH ratio are observed in women with PCOS. The new therapeutic substances could base their action on the inhibition of GABA and kisspeptin stimulation of GnRH neurons [18,44,77,80].

An important aspect of the treatment of patients with PCOS is the sensitization of tissues to insulin. When used in the treatment of type 2 diabetes, metformin is a good drug that increases tissue insulin sensitivity in patients with PCOS. Some researchers show that metformin can also be effective in combating other symptoms of PCOS, such as acne and hirsutism [11,106,107,108,109]. Nevertheless, a long-term use of metformin may lead to versatile complications that include gastrointestinal symptoms (diarrhea, nausea, vomiting and abdominal bloating) and metabolic problems. A possibility to enhance the effect of metformin and reduce its dosage can be provided by administration of inositols. According to the latest research, inositols are similarly effective in combating insulin resistance and the subsequent hyperinsulinemia in the treatment of PCOS [120,121,122,123].

Since inositols are efficient insulin sensitizers, these compounds act on the primal causes of PCOS symptoms on molecular level (namely, they prevent the insulin-resistance which constitutes a key factor in mechanisms of PCOS pathogenesis). Regulation of insulin-mediated processes and preventing excess steroidogenesis predestine the inositols to exert satisfactory therapeutic effects in PCOS patients (both alone and in combination with metformin) [121]. The questions which still require refining in inositol-based therapy are dosage, its effective absorption and large-scale monitoring of the pregnancy success.

The available literature indicates proteins from the GLUT type 4 family as targets for new agents combating insulin resistance in PCOS patients. Over-expressing syntaxin 4 can lead to an enhanced insulin response by enhancing the translocation of GLUT type 4 vesicles to the skeletal muscle cell membrane [113,114,116,117,120]. When looking for new therapeutic substances, one must not forget about natural substances, such as berberine modulating GLUT type 4 expression and naringenin, which increases the concentration of enzymes responsible for scavenging free oxygen radicals and lowering the levels of testosterone and estradiol [117,133].

The research on the mechanisms of PCOS development cannot ignore the influence of inflammatory processes on the development of insulin resistance and the possibility of using SHBG to monitor the course of the disease and as a point of action of new substances [39,86,137]. One should also note the impacts of a proper diet and physical activity on PCOS patients [85,86,109,111,138].

It is difficult to formulate specific guidelines in regard to handling the above-mentioned environmental risk factors for PCOS pathogenesis. Many of the relevant issues can be summarized as lifestyle choices (e.g., avoiding circadian clock disruption). For some factors, however, a top-down regulatory approach may be required, as exemplified by the issue of plasma fluorine levels. In those cases, more strict monitoring and a reevaluation of the acceptable concentration thresholds of various agents may be well-justified.

There is a need to conduct further research to find new drugs and natural substances that could improve the well-being of PCOS patients by alleviating the symptoms of the disease. Some of the inferences presented in this paper refer to the results obtained from animal models (e.g., in case of naringenin or rutin); thus, whether these findings may be extrapolated to human pathophysiology needs to be further investigated.

## Figures and Tables

**Table 1:** Selection of the most important genes involved in PCOS etiology.

Gene Type	Function	Group
CYP11a	Present in all steroid-producing tissues, encodes a cytochrome P450 enzyme that mediates the cleavage of the cholesterol side chain, which is a dominating process in the rate of formation steroid hormones; its role was also confirmed in etiology of hyperandrogenism and PCOS [21,22].	Ovarian and adrenal steroidogenesis
CYP17	Encodes an enzyme cytochrome P450-C17; component of the androgen synthesis pathway, which is dysfunctional in PCOS [23]. Its role in PCOS was also characterized in [24,25].	
CYP19	Encodes important enzymes in androgen synthesis pathways, including cytochrome P450 aromatase. Its role in PCOS was described in [26]. The changes in concentration of P450 was combined with PCOS; for details please see [27]	
CYP21	Encodes an important 21-hydroxylase enzyme involved in synthesis pathways of steroid hormones; reported increased frequency of heterozygosity for mutations in the 21-hydroxylase gene in women with PCOS [28,29].	
LH	Encodes beta subunit of luteinizing hormone. High level of circulating LH is a common biochemical indicator of PCOS [30]; point mutation-Trp8Arg and Ilg15Thr in the gene encoding beta subunit was identified in patients with PCOS [31].	Gonadotropin release regulation
FSHR	Protein encoded by this gene – G-protein coupled receptor is involved in hormonal regulation of gonadal development. Mutation of this gene disrupts the structural conformation of protein and in result causes ovarian hyperstimulation syndrome; the association of follicle-stimulating hormone receptor (FSHR) and PCOS is characterized in [32,33]	
AMH	Encodes the anti-müllerian hormone, it is involved in gonadotropin secretion. The important role of AMH in the pathophysiology of PCOS was characterized in [34,35].	
INSR	Insulin receptor gene on chromosome 19p13.2; Identified region D19S884 was characterized as a fragment of chromosome involved in PCOS [36].	Insulin secretion and action
CAPN10	Encodes Calpain 10 protein, belonging to calcium-dependent cysteine proteases family, which impacts on insulin metabolism and secretion what is the reason for association CAPN10 with PCOS etiology [5,37].	
IRS1, IRS2	Involved in insulin secretion and action; encodes insulin receptor substrates IRS1 and IRS2. Gly972Arg variant of IRS1 was identified more often in women with PCOS [38].	
AR	Mutations in androgen receptors cause the disruption of the respective cellular pathways and in result- hyperandrogenism associated with PCOS.	Steroid hormone action
SHBG	SHBG gene is in 17p13-p12 chromosome and encodes Sex Hormone Binding Globulin, an important biomarker in PCOS risk assessment which binds androgens and other hormones, thus regulates the androgen level in the body [39].	
TNF-alpha	Encodes a cytokine Tumor Necrosis Factor which modulates several biological processes, including immunity and inflammation, obesity and insulin resistance; its role in PCOS has been examined [40].	Chronic inflammation
FTO	Gene encodes fat mass and obesity-associated protein. FTO rs9939609 polymorphism is significantly associated with risk of PCOS [30].	Adipose tissue metabolism

## Abbreviations

The following abbreviations are used in this manuscript:

AKR	aldo-keto reductase
AMH	anti-müllerian hormone
AMP	adenosine monophosphate
AMPK	adenosine monophosphate-activated protein kinase
AQP	aquaporin
BAT	brown adipose tissue
CYP	cytochrome P450
DCI	D-chiro-inositol
DHT	dihydrotestosterone
FSH	follicle stimulating hormone
GABA	γ-aminobutyric acid
GLUT	glucose transporter (protein)
GnRH	gonadotropin-releasing hormone
hCG	human chorionic gonadotropin
HNF-4α	hepatocyte nuclear factor 4 alpha
HSD	hydroxysteroid dehydrogenase
IGF-1	insulin-like growth factor-1
IPG	inositol phosphoglycan
LH	luteinizing hormone
MI	myo-inositol
PCOS	polycystic ovary syndrome
SHBG	sex hormone-binding globulin
